# Towards improvements in foot-and-mouth disease vaccine performance

**DOI:** 10.1186/s13028-020-00519-1

**Published:** 2020-05-20

**Authors:** Graham J. Belsham

**Affiliations:** Department of Veterinary and Animal Sciences, University of Copenhagen, 1870 Frederiksberg C, Denmark

**Keywords:** Capsid assembly, Duration of immunity, Persistent infection, Picornavirus

## Abstract

Foot-and-mouth disease (FMD) remains one of the most economically important infectious diseases of production animals. Six (out of 7 that have been identified) different serotypes of the FMD virus continue to circulate in different parts of the world. Within each serotype there is also extensive diversity as the virus constantly changes. Vaccines need to be “matched” to the outbreak strain, not just to the serotype, to confer protection. Vaccination has been used successfully to assist in the eradication of the disease from Europe but is no longer employed there unless outbreaks occur. Thus the animal population in Europe, as in North America, is fully susceptible to the virus if it is accidentally (or deliberately) introduced. Almost 3 billion doses of the vaccine are made each year to control the disease elsewhere. Current vaccines are produced from chemically inactivated virus that has to be grown, on a large scale, under high containment conditions. The vaccine efficiently prevents disease but the duration of immunity is rather limited (about 6 months) and vaccination does not provide sterile immunity or block the development of carriers. Furthermore, the vaccine is quite unstable and a cold chain needs to be maintained to preserve the efficacy of the vaccine. This can be a challenge in the parts of the world where the disease is endemic. There is a significant interest in developing improved vaccines and significant progress in this direction has been made using a variety of approaches. However, no alternative vaccines are yet available commercially. Improved disease control globally is clearly beneficial to all countries as it reduces the risk of virus incursions into disease free areas.

## Introduction

Foot-and-mouth disease (FMD) remains one of the most feared infectious animal diseases in countries with a highly developed livestock production industry as reviewed in [1–3]. The disease is caused by infection with FMD virus (FMDV), a member of the picornavirus family. The virus infects important domesticated production animals including cattle, pigs, sheep, goats and buffalo plus about 70 species of other cloven-hoofed wildlife animals. The disease is diagnosed based on clinical signs, including high body temperature, excessive salivation, formation of vesicles in and around the mouth and in the inter-digital spaces and on the coronary bands on the feet. Female animals may also have vesicles on the teats. Similar clinical signs can also be caused by other viruses. Hence, in countries that are normally FMD free, it is essential that laboratory analysis is performed on suspected cases (e.g. using real time quantitative RT-PCR). Infected animals lose weight, are prone to secondary infections and the disease can cause long-term loss of productivity, e.g. reduced milk yield. Within infected premises, a very high proportion of the animals often become infected since the virus spreads very easily between animals but there is only low level mortality, mainly due to myocarditis in young animals. In cattle, buffalo and sheep (but not pigs), following the acute stage of infection, a high proportion of animals (e.g. about 50% of cattle) may become persistently infected with low levels of infectious virus present in the oropharynx. These animals are referred to as “carriers” [4–6]. This carrier-state is defined as the maintenance of the virus in the animal for more than 28 days post infection. Different species can carry the virus for months (sheep) or several years (cattle and buffalo). The epidemiological significance of the carrier state is controversial; it has proved impossible to demonstrate, experimentally, transmission of the virus from carrier cattle to naïve cattle by natural, direct, contact. However, transmission from buffalo to cattle has been reported [7]. Recently, it has been demonstrated that direct transfer of oropharyngeal fluid from carrier cattle into the oropharynx of naïve cattle resulted in very efficient infection [8]. Thus, it seems that carrier cattle do constitute a real risk for virus transmission even if that risk is quite low.

While some parts of the world are normally free from FMD, e.g. Europe and North America, it remains endemic in many countries, especially throughout much of Africa and in southern Asia. There has been a considerable improvement in the disease status of South America. There were no reports of clinical disease between the outbreaks in 2013 (in Venezuela) and 2017 (in Colombia) [9], however extensive vaccination is still practiced in various countries in this region which may have masked virus circulation. FMD has been estimated to cause economic losses of about 8–22 billion USD each year [10], in direct and indirect costs within endemic countries. The incursion of FMDV into normally disease free-countries can also have enormous economic consequences. Notably, the large outbreak of FMD that occurred in the U.K. in 2001 is estimated to have cost the country about 10 billion USD [11]. This outbreak affected about 2000 premises, lead to the destruction of over 6 million animals, and lasted for approximately 8 months. The disease also spread from the U.K. into Ireland, France and The Netherlands.

In the 1950s and 60s, very large numbers of FMD outbreaks used to occur each year in Europe [12]. However, with well-organized veterinary services and the extensive use of vaccination (in some countries), the situation improved dramatically and by the 1970s the number of outbreaks of the disease in Europe became very low. In consequence, vaccination against FMD was banned by the European Union (EU) in the early 1990s but emergency vaccination, in the face of an outbreak, has been permitted [13]. One consequence of this approach is that the animal population in Europe is now fully susceptible to the disease, however this greatly facilitates trade in animals and animal products.

No vaccination was used in the U.K. to control the large outbreak of disease in 2001; however, in The Netherlands, vaccination was employed as a control measure to restrict the spread of the disease but all the vaccinated animals were then destroyed [14].

There are seven different serotypes of FMDV known; these are termed O, A, C, SAT (Southern African Territories) 1, 2 and 3 plus Asia-1. There have been no reports, anywhere, of disease due to the serotype C FMDV since 2004, thus this serotype may now be extinct outside of the laboratory [15]. There is little or no cross protection between the serotypes. Thus, animals that have been infected, or vaccinated, with one serotype remain highly susceptible to infection by other serotypes. Indeed, because of the heterogeneity of viruses even within a single serotype, animals vaccinated with one specific strain of the virus may not be protected against infection by another virus of the same serotype. Thus, if vaccination is to be used, then there is a need to match vaccines against strains of the virus that are causing disease in the field, not only at the level of the serotype.

Serotype O FMDV is the most frequently reported form of the virus; it was reported, about 20 years ago, to be responsible for about 70% of the outbreaks globally [16] and this situation remains broadly unchanged [15]. As indicated above, there is significant diversity within serotypes, especially between viruses from different parts of the world. Indeed, with the exception of the Asia-1 serotype, each of the virus serotypes has been classified into different topotypes, based on nucleotide sequence analysis, with distinct geographical distributions [16, 17]. The SAT serotypes and Asia-1 viruses are quite geographically constrained (as their names suggest) but do occasionally move outside of their usual areas, indeed Asia-1 FMDV has reached Greece [18] and SAT 2 FMDV has been present in Egypt in recent years [15]. In contrast, the O and A serotypes (and formerly the C serotype) have had wide geographical distributions.

### Search strategy

This critical review is based mainly on published literature available in PubMed (http://www.ncbi.nlm.nih.gov/pubmed) and builds upon extensive experience of this topic.

### Current FMDV vaccines

Globally, about 2.5 billion doses of FMD vaccine are used annually, mainly in China and South America [10]. Currently, FMD vaccines are normally produced, under high containment conditions, by growing the infectious virus within baby hamster kidney (BHK) cells in suspension culture. The virus particles are chemically inactivated using binary ethyleneimine (BEI), which modifies the viral RNA, then purified to remove non-structural viral proteins. Prior to administration, the vaccine is mixed with an adjuvant (oil or in aqueous form with aluminium hydroxide and saponin).

**Fig. 1:**
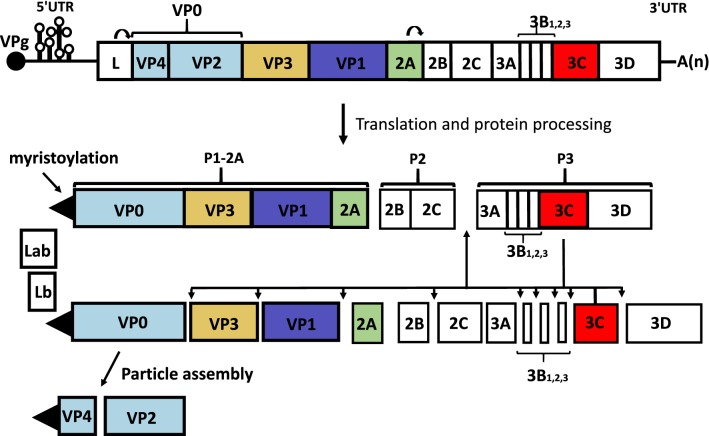
Production and processing of FMDV proteins. The FMDV RNA (top) encodes a large polyprotein that is processed, during and after translation, to a collection of precursors (P1-2A, P2 and P3) and mature products largely through the action of the 3C protease. Many other processing intermediates are made but are not shown. The cleavage of the myristoylated VP0 to VP4 and VP2 occurs during particle assembly. The myristoylation of the N-terminal glycine of the capsid precursor P1-2A is achieved by a host cell system. The two forms of the Leader protease (Lab and Lb) cleave themselves from the capsid precursor. The 5′ UTR includes extensive secondary structure, one region is the internal ribosome entry site (IRES) required for initiation of protein synthesis on the viral RNA

Infectious FMDV particles are roughly spherical (about 25–30 nm in diameter) and have a protein shell, which contains 60 copies of each of the 4 different structural proteins VP1–VP4, that encloses a single copy of the positive sense RNA genome (Fig. 1). The FMDV RNA genome includes one, large, open reading frame and acts like a mRNA encoding a single polyprotein (Fig. 1). However, the intact polyprotein is never observed since during, and after, translation, the polypeptide chain is cleaved, mainly by virus-encoded proteases (L and 3C), to produce a variety of primary precursors (P1-2A, P2 and P3), which are further processed to make a collection of 15 mature virus proteins (see below). The four structural proteins (VP1–VP4) are generated from the P1-2A precursor while the non-structural proteins (NSPs), which are mainly involved in protein processing, RNA replication and anti-host defense mechanisms, are derived from the P2 and P3 precursors.

#### Virus particle assembly

**Fig. 2:**
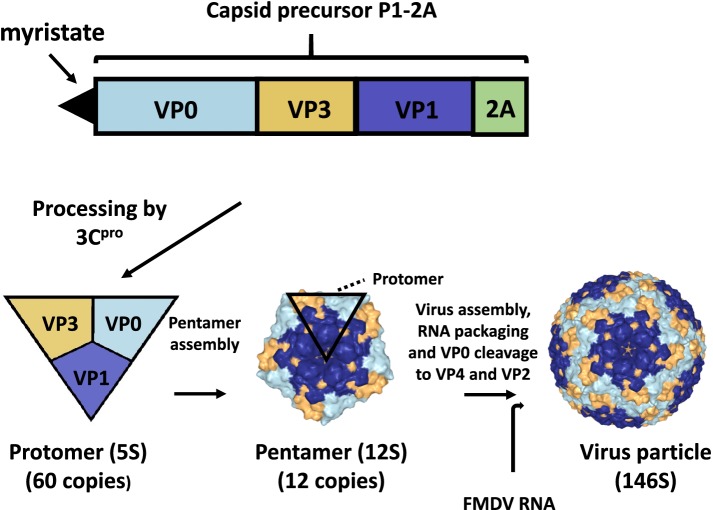
The assembly of FMDV particles. The myristoylated capsid precursor protein (P1-2A) is cleaved by the 3C protease to form protomers (5S) consisting of VP0, VP3 and VP1. Five of these protomers assemble into pentamers (12S) and 12 of these combine to form the near spherical capsid particles containing 60 copies of each of the viral capsid proteins. When RNA is packaged, then infectious particles are formed (146S) but non-infectious empty capsid particles (70S), without any viral RNA, can also be made (not shown). The cleavage of VP0 to VP4 and VP2 (see Fig. 1) accompanies the process of particle assembly and, at least for FMDV, does not require the presence of viral RNA

The FMDV particles are assembled through a series of steps. The various intermediates are identified from their sedimentation characteristics (S value) during sucrose gradient centrifugation. This type of analysis may put constraints on what can be detected since the intermediates have to be stable for the duration of the analytical procedure. The capsid precursor P1-2A (Fig. 2) is modified at its N-terminus by the addition of a myristate (C14) group through the action of a cellular myristoylation system [19, 20]. It is also cleaved by the 3C protease (3C^pro^) to produce VP0, VP3, VP1 plus the 2A peptide, the latter is not usually incorporated into the virus but it can be, as VP1-2A, if the cleavage junction is modified [21]. The capsid proteins remain associated with each other within a protomer (5S) and 5 protomers assemble together to form a pentamer (12S). Subsequently, twelve pentamers assemble, with the viral genome, to form the intact virus particle (146S). During this final assembly step, the VP0 is cleaved to VP4 plus VP2 by an unknown mechanism. Assembly of pentamers into non-infectious empty capsid particles (70S), lacking the RNA genome, can also occur. At least with FMDV, the cleavage of VP0 seems to be dependent on the particle assembly process rather than the presence of viral RNA [22, 23], thus assembled empty capsid particles have been found to contain VP2. The production of empty capsid particles seems to vary in efficiency between virus strains; serotype A empty capsid particles are readily formed within FMDV-infected cells [24] but are less abundant with most strains of serotype O virus. This may have consequences for the efficiency with which empty capsid particles, of different strains/serotypes, can be generated using recombinant protein expression systems [25].

The intact virus particles (or empty capsid particles) are much more immunogenic than individual virus components or even partially assembled virus particles (e.g. pentamers) or their breakdown products [26]. Thus, it is important to ensure that vaccines contain a high level of intact particles when produced and that they are stored under conditions that preserve this (including use of a cold chain during vaccination campaigns). Systems to detect and quantify the presence of intact particles (of certain serotypes) have been developed [27, 28].

### Limitations of current FMD vaccines

As indicated above, the vaccine has been proven to be very effective in helping to control the disease in Europe, in conjunction with other control measures including animal movement controls, but the current FMD vaccines are far from perfect. Vaccination prevents the appearance of disease but limited virus replication, especially in the oropharynx, can still occur in these animals and this can be sufficient to allow those vaccinated animals, which do get infected, to become carriers and thus harbor the infectious virus for a long period of time.

If the virus particles are purified away from the non-structural proteins (NSPs) during manufacture, then it is possible to distinguish, by serology, between animals that have been vaccinated against FMD but not infected and those that have been infected with the virus (the DIVA concept). Both the virus infection and the inactivated vaccine induce the production of antibodies against the capsid proteins. In contrast, antibodies against the NSPs should only be generated by infection (although multiple vaccinations with purified vaccines may still elicit an anti-NSP response). A variety of assays are available to detect antibodies against the NSPs [29]. However, if a vaccinated animal becomes infected (albeit without disease) then the level of virus replication in these carrier animals may be insufficient to generate an immune response to the NSPs even though the infectious virus is still carried by the animals [29, 30].

Some of the limitations of the current FMD vaccines are discussed further below. Some of these limitations reflect the nature of the vaccine while others reflect the biology of the virus, in particular its rapid evolution including incremental genetic drift and more radical recombination events.

### FMDV biology

The FMDV genomic RNA is positive sense and, when introduced into the cytoplasm of cells, is sufficient to initiate infection [31, 32]. The RNA sequence contains a single, large, open reading frame (ca. 7000 nucleotides (nt)) encoding a polyprotein that is flanked by a long (ca. 1300 nt) 5′-untranslated region (UTR) and a much shorter 3′-UTR (ca. 90 nt) followed by a poly(A) tail (Fig. 1). Initially the genomic RNA has to function like a mRNA; it encodes a polyprotein of about 2300 amino acids. However, the complete polyprotein is never made as it is cleaved during, and after, synthesis into a variety of precursors that are further processed to make 15 different mature proteins (Lab, Lb, VP4, VP2, VP3, VP1, 2A, 2B, 2C, 3A, 3B1, 3B2, 3B3, 3C and 3D) (see Fig. 1). These include the 4 distinct structural proteins (VP1, VP2, VP3 and VP4) that form the virus capsid (Fig. 2). This protein shell (with 60 copies of each capsid protein per particle) protects the viral RNA when the virus is outside of a cell and also facilitates delivery of the viral RNA to the cytoplasm of a new cell so that a new round of infection can occur. The capsids bind to cell surface integrin receptors [33] and, following virus entry, the mild acidification that occurs within endosomes [34] is sufficient to breakdown the acid-labile virus capsid and permit the release of the viral RNA so that translation can commence. The eleven non-structural proteins, include two different forms of the Leader proteinase (termed Lab and Lb), resulting from initiation of translation at two different AUG codons [35] and three non-identical forms of 3B (termed 3B1, 3B2 and 3B3). These 3 short peptides are also known as VPg (Virus Protein genome linked) as they are covalently linked to the 5′-terminus of all the newly synthesized viral RNA (Fig. 1). Each VPg can act as the substrate for uridylylation to form VPgpUpU [36], which then acts as the primer for RNA synthesis. The 3C protease (3C^pro^) is responsible for most of the proteolytic processing events within the FMDV polyprotein while the 3D protein is the RNA dependent RNA polymerase, termed 3D^pol^. The properties and functions of the different virus encoded proteins have been reviewed in detail separately [37, 38] and will not be repeated here.

#### Viral RNA replication is error prone

In addition to acting as a mRNA, the FMDV genome also acts as the template for RNA replication. Thus, for at least one molecule within each infected cell, translation of the input viral RNA has to cease to allow the synthesis of a negative sense RNA. Note, during protein synthesis, the ribosomes move along the RNA in a 5′ to 3′ direction while, for RNA replication, the RNA polymerase begins synthesis of the negative strand at the 3′-terminus of the RNA; these processes cannot occur simultaneously on the same molecule. The newly synthesized negative strand is then used as the template for the production of, many more, positive-sense RNA copies that can be used for translation (for the production of viral proteins), or as a template for further negative sense RNA production or packaged into new virus particles (Fig. 2).

The replication of viral RNA occurs in structures, derived from intracellular membranes [39], termed replication complexes or replication organelles which contain a number of host and viral proteins (including 2C and 3D^pol^) that are required for the process [40]. The replication of the viral RNA is highly error prone, i.e. incorrect nt are incorporated into the RNA copies. Assessments of the error rate of the RNA polymerase suggest that, on average, about one error is made for every 10,000 nt that are synthesized [41]. This means that it can be expected that nearly every FMDV genome has at least one error since about 17,000 nt have to be copied to make one new genomic RNA molecule (after copying both a positive and a negative strand). There is no known proof reading mechanism in picornaviruses; thus, the total viral RNA population represents a pool of closely related sequences; this pool is known as a quasi-species [42]. Modifications to the fidelity (that either increase or decrease the error rate) of the 3D^pol^ from picornaviruses reduce the “fitness” of the virus [43]. Thus, it appears that for these viruses, there is a balance between the need to maintain a fully functional RNA sequence and the requirement to be able to adapt rapidly to new conditions. As a result of this continuous generation of errors, the virus population is always evolving. However, it should be noted that the “consensus” sequence (i.e. corresponding to the predominant nt present at each position in the genome) of the virus population will only change relatively slowly (compared to the error rate) when some of the errors become fixed (i.e. they become predominant), presumably because they confer some selective advantage. The consensus sequence of the FMDV population changes at 0.5–1.0% of the genome per year [44]. This represents about 40–80 nt per year or around 1–2 nt changes per week. These differences in sequence can potentially modify the biology of the virus, (e.g. in its antigenicity or speed of replication) but can also be useful for tracing the spread of viruses during disease outbreaks [44, 45].

It can be expected that RNA polymerase errors will occur throughout the entire viral genome and evidence derived from the analysis of different virus isolates obtained from the outbreak in the U.K. in 2001, starting from a single source and spreading within unvaccinated animals, suggests that this is indeed the case [44]. From a collection of nearly 200 different nt substitutions identified within 23 different full-length virus sequences, some 28 changes were within the non-coding regions (i.e. about 14%, which is close to the proportion of the genome that these non-coding regions constitute within the genome). Within the coding region for the whole polyprotein, a major proportion of the changes observed were synonymous and only 40 (20%) of the changes modified the encoded protein sequence. Thus, it seems clear that selection to retain the amino acid sequence is high. Amino acid changes that modify the function of the protein are usually going to be deleterious and thus will not be maintained. However, some regions of the genome are much more tolerant to change than others. Clearly, within a vaccinated population there can be selection pressure for variants that have modified antigenicity.

### FMDV sequence diversity

From comparing over 100 strains of FMDV, including representatives of all 7 serotypes, Carrillo et al. [46] found that within the 5′-UTR, the average nt identity between all serotypes is over 80% and for the entire polyprotein coding region (ca. 7000 nt) the level of sequence identity between any two virus isolates was at least 73%. However, the VP1 coding region (about 639 nt) is substantially more variable between strains than most of the polyprotein coding sequence and shows only about 50–70% nt identity between all serotypes [47]. The VP1 itself has the lowest proportion (24%) of invariant amino acids among the different products derived from the polyprotein [46]. Presumably this reflects the ability of certain residues to accept change (e.g. in loop regions connecting structural elements within the surface exposed capsid proteins VP1, VP2 and VP3) and selection pressure resulting from immune responses to these exposed features of the virus particle. The capsid protein VP4, which is entirely internal within the intact particle, is much less variable (81% invariant residues, [46]).

Some surface exposed parts of the capsid proteins (including the antigenic sites, see below) are clearly able to change extensively. However, even within the VP1 capsid protein, there are highly conserved motifs, e.g. the RGD (Arg-Gly-Asp) motif. This is critical for interaction with the cellular integrin receptors and therefore required for virus attachment and entry into cells [33]. Similarly, a very highly conserved YCPRP (Tyr-Cys-Pro-Arg-Pro) motif near the C-terminus of FMDV VP1 was apparent from the alignments performed by Carrillo et al. [46] but its significance has only recently been recognized [48, 49]. This motif is required for processing of the capsid precursor P1-2A by the 3C^pro^ and is also highly conserved between different picornavirus genera (e.g. WCPRP in enteroviruses and FCPRP in cardioviruses; note, W (Trp) and F (Phe) are aromatic amino acids like Y (Tyr)). No doubt, errors in the nt sequences encoding these conserved amino acids do occur but presumably the resultant viruses (if viable at all) are not efficiently propagated and hence such variants are not maintained within the virus population.

### RNA recombination

In addition to the gradual accumulation of nt changes described above, a more dramatic form of genome evolution, involving RNA recombination, can also occur within picornavirus genomes. During the process of picornavirus RNA replication, it is possible for the RNA polymerase (3D^pol^) to switch from copying one positive strand template to another [50], by a so-called “copy-choice” mechanism. This process can result in the formation of “chimeric” genomes, e.g. with the capsid coding sequences derived from one parental virus and the rest of the genome derived from a different strain of the virus [51, 52]. Thus, recombination can change the serotype of the virus. The switching of templates during RNA replication may occur very frequently during RNA replication but if all the genomes within a single cell are all very closely related then this will not have any significant effect on the outcome of the RNA replication process and will be hard to detect. However, if a cell is co-infected with two genetically distinguishable genomes then a novel chimeric genome can be produced by the recombination that may, or may not, be viable. If it is viable, then it can have different properties from each of the parental virus strains and, under appropriate conditions, may replicate preferentially. Clearly, the production and detection of a novel recombinant virus in the field requires that an animal is co-infected with distinguishable strains of the virus. In some parts of the world, multiple serotypes of the virus frequently co-circulate and evidence for inter-serotypic recombination in FMDV in the field has been described [51, 53, 54]. Evidence for recombination between different lineages of the same serotype has also been reported [55]. The identification of recombination is facilitated if (near) full-length genome sequences are generated so that the genetic relationships between different parts of the genome to other viruses can be established [54].

#### How does recombination occur?

As indicated above, detecting recombination requires that an animal is co-infected with two distinct strains of FMDV. Indeed, the same cells within the host need to be infected at the same time by each virus so that the viral RNA polymerase can switch between the two different FMDV RNA templates. When an animal is infected with FMDV, there is usually a fairly short, acute phase of infection [1, 56]. A high level of viremia is apparent for a few days and vesicular lesions, containing high levels of virus, are observed. The infection subsides as a protective immune response is generated and the vesicles heal. However, as indicated above, many infected animals (ca. 50% of cattle) do not completely clear the infection and maintain a low level of infectious virus within the oropharynx for months or even years. It should be noted that pigs do not become carriers [57]. It is not yet established whether recombination between FMDVs occurs when the parental viruses are each causing an acute infection within an animal simultaneously or if the re-infection of a “carrier” animal with a different virus is capable of allowing co-infection of cells with the two different strains of virus. The extremely high level of virus present within an acutely infected animal potentially makes it easier for individual cells within the host to be co-infected but, clearly, this imposes a fairly narrow time window for the co-infection of the cells to occur. However, re-infection of a “carrier” animal with a different strain of virus could occur at a time interval of weeks, months or even years after the primary infection. If carrier status is the key to recombination, under field conditions, then it should not happen in pigs. In general, it is not known in which species any recombinant event has occurred but a recent study provided evidence for recombination between different variants of SAT 1 FMDV within experimentally infected African buffalo (*Syncerus caffer*) [58]. Thus, it should be possible to design experiments to determine if recombination between FMDVs can occur in pigs.

### Antigenic diversity of FMDV and vaccine selection

The existence of 7 serotypes of FMDV clearly indicates that the genetic diversity displayed by the virus results in antigenic diversity as well. The antigenic properties of the virus are dependent on the surface exposed residues of VP1, VP2 and VP3 (note VP4 is entirely internal within the virus particle, [59]).

Antigenic matching between viruses is important for the selection of the most appropriate vaccine to protect against a strain of FMDV causing disease. In principle, this could be performed by conducting a vaccination trial within natural host animals with potential vaccines and then challenging the animals with the outbreak strain. This would be very time consuming and expensive. In practice, in vitro neutralization assays are commonly used to determine whether antisera generated by particular vaccines are able to efficiently neutralize the outbreak strain. Unfortunately, such assays can be rather poorly reproducible [60].

#### Vaccine potency testing

The usual potency testing of FMDV vaccines involves the inoculation of a necessarily small number of animals with different doses of the vaccine and then challenge, by needle inoculation, at 21 or 28 days post vaccination, with an appropriate (normally homologous) virus strain [61]. While this test has some useful features, in terms of standardization, it also has certain limitations. For example, the test does not give information about the ability of the vaccine to protect against non-homologous virus strains or to protect against a more usual form of virus challenge, i.e. by exposure to an infected animal, and only relates to a single time point post-vaccination with limited precision due to the small groups of animals. A detailed and comprehensive review of the issues relating to evaluating, in the laboratory, the protection conferred by specific FMD vaccines has been published by Paton et al. [60]. Some field studies to examine the effectiveness of FMD vaccines against a specific virus threat have also been reported [62]. Furthermore, a review considering the design of studies to assess the efficacy of vaccines in the field has been published recently [63].

Monoclonal antibodies (Mabs) raised against FMDV can be used as standardized reagents to assess the antigenicity of different viruses. Such Mabs that neutralize virus infectivity have been used to select for neutralization resistant mutants of FMDV using a range of different serotypes of the virus. By sequencing the selected, neutralization resistant, viruses, it is possible to identify key surface exposed residues in FMDVs that are important for the antigenicity of the virus. Using this approach, multiple, independent antigenic sites have been identified. In general, these antigenic sites are located on surface exposed loops and each of the surface exposed capsid proteins make some contribution towards them [64–71]. Interestingly, sequential selection of a serotype O virus that was resistant to neutralization at each of 4 separate antigenic sites still resulted in a virus that was efficiently neutralized by a serotype O-specific polyclonal antiserum [72]. Thus, the relationship between the antigenic sites, defined by selection with murine Mabs, and the epitopes recognized by sera from natural host animals is not simple. It should be noted, however, that the antigenic sites identified using Mabs do correspond to regions of high sequence variability within field viruses suggesting these regions have changed due to antigenic pressure. These studies do not, however, necessarily, reveal all residues bound by the Mab. For example, if a Mab binds to a region of the virus that has to be maintained for virus viability (e.g. to bind to the integrin receptor) then clearly, the Mab-resistant mutants selected will not be modified at that residue. Indeed, an analysis of the interaction between a particular Mab (D9) with the intact serotype O virus, using cryo-electron microscopy, revealed interaction of the Mab with residue D147 within VP1 (part of the RGDL motif required for integrin binding) that had not been identified previously from sequencing of neutralization resistant mutants [73]. However, substitution of the adjacent residue L148 in VP1 had been shown to result in strong resistance to neutralization by the Mab D9 [64, 66], thus it is not surprising that residue D147 also interacts with the antibody.

In principle, it is possible to assess how an outbreak strain of FMDV corresponds to known vaccine strains in these key antigenic regions of the virus capsid either through measuring reactivity with mapped Mabs or from sequence determination [74–76]. However, the ability of a vaccine to efficiently confer protection against virus challenge in the field undoubtedly depends on many factors, in addition to the actual “match” between the outbreak virus and the vaccine. An analysis of the ability of a range of different serotype O vaccine strains to combat multiple serotype O viruses circulating in southern Asia has been published [77]. It is apparent that the overall ability of a vaccine to induce neutralizing antibodies (as measured by in vitro virus neutralization tests) must be important but within the host, it is possible for non-neutralizing antibodies to reduce the level of circulating virus as well. The strength of the immune response to the vaccine will also depend on the amount of antigen that is in the vaccine and its integrity as intact particles. High potency vaccines can generate protective immunity within a few days [78] and this immunity can last for at least 6 months from a single dose [79]. However, the duration of immunity generated by the current inactivated vaccines (using a standard dose) is often rather limited. In endemic regions, it is frequently necessary to re-vaccinate animals at least two times per year [80] to maintain protection. Thus, the timing between vaccination and the exposure to virus challenge and also, perhaps, the actual level of virus encountered in the field can be important.

It is worth noting that some FMD vaccines, e.g. the widely used O1 Manisa vaccine, are based on viruses that circulated a long time ago (O1 Manisa was isolated in 1969). However, despite the genetic diversity of the virus, high potency vaccines based on this strain are still able to provide protection against heterologous serotype O strains [81, 82]. Similarly, intra-serotypic protection against very diverse serotype A strains has been demonstrated with high payload serotype A vaccines despite low levels of antigenic “match” [83].

### Prospects for improved FMD vaccines

**Fig. 3:**
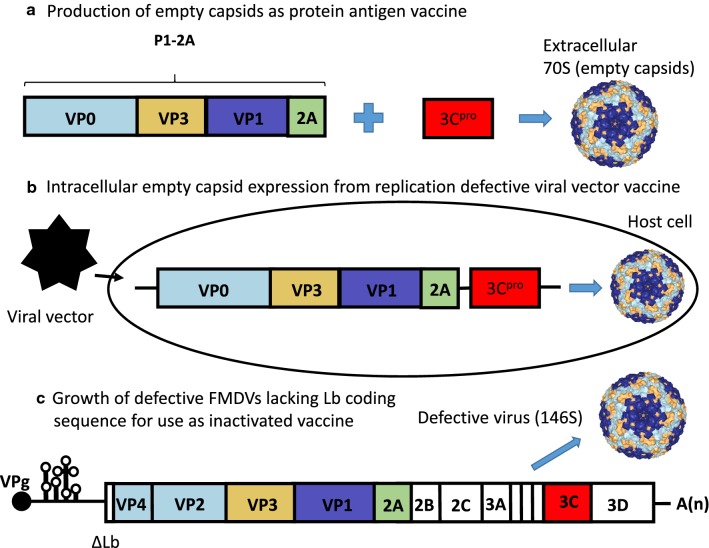
Alternative strategies to produce novel, safe, FMDV vaccines. **a** The production of 70S empty capsid particles by the co-expression of the FMDV P1-2A capsid precursor plus the 3C^pro^ has been achieved using baculovirus and vaccinia virus expression systems. The purified non-infectious particles can be used as a vaccine. **b** Defective viral vectors (e.g. human adenovirus Ad5 or Semliki Forest virus vectors) have been used to express the P1-2A plus 3C^pro^ within mammalian cells. These viral vector vaccines can infect the host’s cells but cannot spread within the host. The FMDV P1-2A + 3C proteins are expressed from the vector within the infected cells and thus produce intracellular empty capsid particles. **c** Defective FMDVs, lacking the Lb coding sequence (c.f. Fig. 1), have been shown to be attenuated in animals and thus can be used to grow FMDV antigen in BHK cells more safely, as infectious virus. It is still expected that the virus will be inactivated prior to use as vaccine, as for current vaccines

A variety of reviews have described the need for improved FMD vaccines [80, 84, 85] and the current status of developments towards improved FMD vaccines [38, 86]. For a detailed description of the major current strategies for the development of new FMDV vaccines the reader is referred to these reviews. In brief, there are just a few main approaches that have continued attention. These are outlined in Fig. 3. Essentially each approach is aimed at producing virus-like particles that display the spectrum of antigenic sites that are present on the virus particle itself. Attempts to use individual capsid proteins or synthetic peptides as candidate vaccines have proven unsuccessful. The main focus is now on three types of system which are:

- The production of non-infectious empty capsid particles, essentially by the co-expression of the myristoylated capsid precursor P1-2A with the 3C^pro^ (Fig. 3a) [25, 87, 88].
  The virus vector systems (e.g. vaccinia virus or baculovirus) used to express empty capsid particles in cell culture do not rely on the ability of the FMDV particles to be able to initiate an infection and this allows modification of the capsid proteins to enhance the stability of the assembled particles [88, 89]. These systems also offer the potential for production of FMDV antigens without the need for high containment facilities. However, it seems likely that vaccines based on these products will continue to suffer from some of the same issues as the current inactivated vaccines, e.g. short duration of immunity, lack of sterile protection with the possibility of carrier animal production.
- Use of a replication defective viral vectors to express FMDV empty capsids (Fig. 3b) [90, 91].
  This approach differs from the non-infectious systems described above in that the defective virus vectors are able to infect cells within the host (but do not spread) and thus the FMDV products are produced within the cells of the recipient animals. This can allow a broader range of immune responses to be mounted by the host than are generated by an extracellular protein antigen. The system based on the human adenovirus vector (Ad5) has received “conditional licensing” in the USA that, for the first time, allows production of FMDV vaccine on the US mainland. Currently, high doses of this vaccine are required to achieve protection and, perhaps surprisingly, the duration of protective immunity also declines after about 6 months [92] while the continued presence of anti-adenovirus antibodies may preclude re-vaccination. This may limit the utility of these vaccines to emergency use to combat an outbreak in countries that are usually disease free. The alphavirus system, as used by Gullberg et al. [91], is rather complex to produce but has the advantage that the alphavirus vector, based on an RNA genome [93], replicates solely within the cytoplasm of cells, as does FMDV, and thus issues related to RNA modifications within the nucleus (e.g. splicing, as occurs with the Ad5 vectors) are not relevant.
- Development of modified FMDV strains that are fully attenuated in animals but can be grown efficiently in cell culture (e.g. based on the forms of the virus that lack the coding region for Lb (Fig. 3c) [94].
  It is possible to precisely delete the Lb coding region from FMDV virus without loss of viability within BHK cells (e.g. [94, 95]). However, these mutant viruses are highly attenuated in animals and cannot easily revert to virulence. Thus, these attenuated FMD viruses represent a significantly safer source of FMDV antigen than current vaccine strains. In the unlikely event that escape of the virus from a production facility occurred, the virus would not be able to infect animals and then be transmitted by them. It seems likely that such products could be produced at lower levels of containment than are required for conventional FMDV vaccine but they will have similar properties and limitations as the conventional vaccine. Presumably, the attenuated particles would be chemically inactivated and purified prior to use, as with conventional FMD vaccine viruses.

## Conclusions

Improved knowledge of FMDV biology has allowed the design of novel candidate vaccines using a variety of approaches. Several, distinct systems are still being actively studied but, currently, only the conventional, inactivated FMDV vaccines, essentially developed in the 1960′s, are available for widespread use to combat the disease. The apparent disappearance of serotype C FMDV globally and the usual freedom from disease in Europe together with the much improved FMD situation in South America demonstrate that existing disease control measures, including use of current vaccines, can be effective. In endemic settings, it is clearly important to ensure that confidence in existing vaccines is maintained, or strengthened. Tests to ascertain vaccine quality, both during production and after storage/transportation should be performed. Poor vaccine effectiveness may not only result from a poor match between the vaccine and the circulating virus.

There remains significant interest in developing better FMD vaccines as the disease is still a major problem in many countries and represents a huge barrier to trade in animals and their products for these countries. Improved disease control globally is clearly beneficial to all countries as it reduces the risk of virus incursions into disease-free areas that can have enormous financial consequences.

## Data Availability

All information reviewed in this review article is published.
